# Exploring *Psilocybe* spp. mycelium and fruiting body chemistry for potential therapeutic compounds

**DOI:** 10.3389/ffunb.2023.1295223

**Published:** 2023-11-29

**Authors:** Adam Waldbillig, Maria Baranova, Sarah Neumann, Jonathan Andrade, Sharan Sidhu

**Affiliations:** Numinus Bioscience, Nanaimo, BC, Canada

**Keywords:** metabolomics, *Psilocybe*, mycelia, multivariate analysis, culture methods, PCA, fruiting body, magic mushrooms

## Abstract

*Psilocybe* mushrooms, otherwise known as “magic” mushrooms, owe their psychedelic effect to psilocin, a serotonin subtype 2A (5-HT_2A_) receptor agonist and metabolite of psilocybin, the primary indole alkaloid found in *Psilocybe* species. Metabolomics is an advanced fingerprinting tool that can be utilized to identify the differences among fungal life stages that may otherwise be unaccounted for. In this study, by using targeted and untargeted (metabolomic) multivariate analysis, we demonstrate that the chemical composition of *Psilocybe* differs among mycelia, grain mycelia, and fruiting bodies. The preferential accumulation of psilocybin, baeocystin, tryptophan, ergothioneine, and phenylethylamine in fruiting bodies differentiated them from mycelia; however, the levels of alpha-glycerylphosphorylcholine (α-GPC), *N-*acetylglucosamine, and trimethylglycine were found to be proportionally higher in mycelia than in fruiting bodies based on Pareto-scaled data. Considering the wealth of compounds with therapeutic potential that have been isolated from various fungal genera, it would be pertinent to study the compounds found in *Psilocybe* mycelia as potential naturally derived therapeutic targets.

## Introduction

Psilocybin and psilocin are the primary hallucinogenic alkaloids found in psychedelic mushrooms (Wieczorek et al., 2015) from the basidiomycete genera *Psilocybe*, *Inocybe*, *Panaeolus*, *Gymnopilus*, *Copelandia*, *Hyboloma*, *Pluteus*, *Conocybe*, and *Panaeolina* [also referred to as hallucinogenic, entheogenic, “magic”, medicinal, neurotropic, psychoactive, sacred, and saint mushrooms (Guzmán, 2008)]. Psilocin exerts a neurologic effect by binding to serotonin subtype 2A receptors (5-HT_2A_) and producing neuropsychological effects, including oceanic boundlessness, anxious ego dissolution, visionary restructuring, auditory alterations, and reduction of vigilance; collectively, these comprise the “psychedelic experience” (Hasler et al., 2004). On oral administration in humans, psilocybin is rapidly converted to psilocin via intestinal alkaline phosphatase, where nearly 100% of the bioactive molecule is found in circulation 30 min to 6 h after ingestion (Dinis-Oliveira, 2017). Psilocin is able to pass through the blood–brain barrier (Dinis-Oliveira, 2017) and bind to 5-HT_2A_ receptors in the central nervous system with nanomolar affinity (Rickli et al., 2016), leading to downstream hallucinogenic effects (López-Giménez and González-Maeso, 2017).

**Figure 1:**
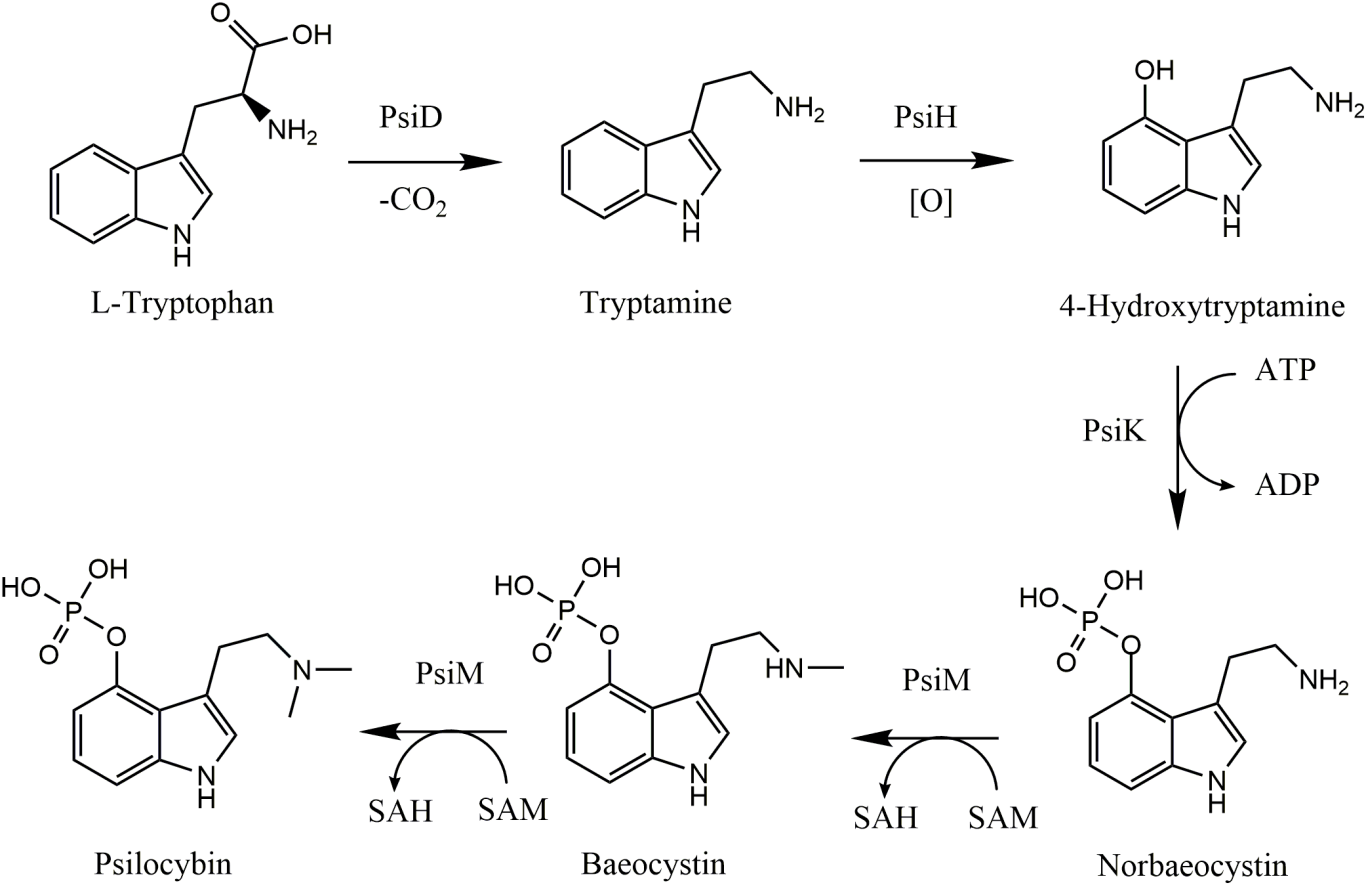
Biosynthesis pathway of psilocybin in *Psilocybe cubensis*. (Adapted from Fricke et al., 2017). PsiD, psilocybin decarboxylase; PsiH, psilocybin hydroxylase; PsiM, psilocybin methyl transferase; PsiK, psilocybin kinase; SAM, S-adenosyl-L-methionine; SAH, S-adenosyl homocysteine.

Psilocybin is synthesized using tryptophan derived either directly from amino acids or from the shikimate acid pathway as its primary substrate, with the latter being the most common in both fruiting bodies and mycelia (Mahmood, 2013). In brief, L-tryptophan is decarboxylated to tryptamine by the psilocybin decarboxylase (PsiD) enzyme where tryptamine is then hydroxylated at position 4 to yield 4-hydroxy-tryptamine by psilocybin hydroxylase (PsiH). Subsequently, 4-hydroxy-tryptamine may be phosphorylated by psilocybin kinase to form norbaeocystin, which is then twice-methylated at the amine group by the psilocybin methyltransferase enzyme to form psilocybin (PsiM) (Fricke et al., 2017; Torrens-Spence et al., 2018) (**Figure 1**). Further methylation of the amine group by PsiM yields aeruginascin, a trimethyl ammonium analog of psilocybin found originally in *Inocybe aeruginascens* (Jensen et al., 2006). The above-mentioned indole alkaloids may exhibit biological activity and have been detected in samples of *Psilocybe cubensis.*

Each stage of the fungal life cycle creates a unique fingerprint of fungal metabolites which may have their own unique therapeutic potential. For example, *Hericium erinaceus* (lion’s mane) mycelia preferentially expresses p450 enzymes involved in the synthesis of diterpenoid “erinacines” (Li et al., 2018), whereas fruiting bodies exhibit higher concentrations of polyketide “hericenones”, but a low erinacine content (Ma et al., 2010). Erinacines have been cited as being anti-inflammatory and reactive oxygen species scavengers, whereas hericenones are promoters of nerve growth factor (NGF) secretion and neurite outgrowth (Phan et al., 2014). Mycelium has been targeted for the development of pharmaceuticals with culture broths as a means of increasing fungal biomass for the purpose of isolating bioactive compounds (Toth et al., 1983; Ikekawa et al., 1985; Kawagishi et al., 1996; Okuyama et al., 2004; Park et al., 2004; Mygind et al., 2005; Kawagishi et al., 2006; Jung et al., 2008; Singh et al., 2011; John et al., 2013; Phan et al., 2015). Some examples of these compounds are an antibiotic compound from *Pseudoplectania nigrella* (Mygind et al., 2005), immunomodulatory compounds from *Aspergillus nidulans* (Singh et al., 2011), and an antitumor agent isolated from *Flammulina velutipes* (Ikekawa et al., 1985).

The multivariate analysis of metabolomic data performed on *Ganoderma lucidum* (reishi or lingzhi) revealed the differential accumulation of metabolites, with 48, 25, 364, and 25 metabolites being unique to the fermentation broth, mycelium, fruiting body, and spore powder, respectively (Xie et al., 2020). Several cultivation methods, substrates, and media may be utilized to produce the fungal material, with expected differences found in the metabolomic profiles. In this study, we analyze the difference between liquid culture and grain spawn substrate for mycelial colonization. Although the genome of *P. cubensis* has recently been drafted, with 32 contigs mapping 97.6% of the genome by benchmarking universal single-copy orthologs (BUSCO) completeness score (McKernan et al., 2021), to date, no metabolomic mapping of *Psilocybe* spp. growth stages has been published. *Psilocybe* spp. mycelia have markedly lower concentrations of psilocybin and psilocin (Gartz and Moller, 1989; Gotvaldová et al., 2021); however, unique compounds were detected in a sample of mycelia when compared with fruiting bodies of the same species using ultra-high-pressure liquid chromatography coupled with mass spectrometry (UPLC-MS) data (Bradshaw Alexander et al., 2022), and, therefore, a more comprehensive analysis of targeted and untargeted fungal metabolite profiles from both fruiting body and mycelial datasets is needed.

In this article, we employed ultra-high-pressure liquid chromatography coupled with ultraviolet and visible light detector (UHPLC-UV/VIS; TSQ Altis™) and ultra-high-pressure liquid chromatography coupled with high-resolution mass spectrometry (UHPLC-HRMS; Orbitrap Exploris™) data coupled with multivariate analysis to compare the targeted and untargeted (metabolomic) chemical profiles of *Psilocybe* spp. mycelia grown in liquid culture, mycelia grown on rye grain substrate, and fruiting bodies harvested from bulk substrate. Evidence of the preferential accumulation of indole alkaloids and also the resulting correlations among other fungal metabolites in mycelial samples vs. fruiting bodies is essential in not only the development of *Psilocybe* fruiting bodies in mycotherapy, but also that of *Psilocybe* mycelia as a lower psychedelic potential alternative.

## Materials and methods

### Sample collection

All fungal strains originated as externally sourced spore prints. The spores were germinated and the dikaryotic mycelium was isolated to either single-sector isolates (P-AZ-1, 5051-B.1, P-CY-1, BP-STAR, and BP-A-STAR) or multi-strain isolates (BP-A-MUL, BP-C-MUL, AV-MUL, and LK-MUL). The strains P-AZ-1, P-CY-1, BP-STAR, BP-A-STAR, BP-A-MUL, and BP-C-MUL were identified through the amplification and Sanger sequencing of the ITS1–5.8S–ITS2 region. DNA extraction was performed at Numinus Bioscience using the Invitrogen™ PureLink™ Genomic Plant DNA Purification Kit, and amplification and sequencing was performed at the Slot Laboratory at Ohio State University, Columbus, OH, USA. The strains AV-MUL, LK-MUL, and 5051-B.1 were identified by confirming the morphological characteristics of the fruiting bodies. All fungal strains were preserved as mycelium cultures stored at 4°C in our parental cell line library.

The mycelial cultures were grown on solid yeast malt extract agar (YMEA) or potato dextrose agar (PDA) in 10-cm Petri dishes, and the mycelia showed no preference toward either substrate. The strains were incubated at 26°C (*P. cubensis*) or 21°C (*Psilocybe allenii* and *Psilocybe cyanescens*) until fully colonized. The cultures were visually inspected for sectoring and, in some cases, continuously subcultured until a single-sector isolate was achieved, while in other cases, cultures were not subcultured and were kept as multi-strain isolates. The mycelia and agar slices were transferred to sterilized rye kernel grain spawn jars and left to fully colonize at 26°C. Once fully colonized, the grain spawn jars were used to inoculate grain spawn bags and left to fully colonize at 26°C. These fully colonized spawn bags were then used to inoculate the bulk substrate bags at a ratio of 1 : 3. On full colonization of the bulk substrate bags, fruiting and harvesting occurred. The fruiting bodies were considered mature and ready for harvest once the veil was separated from the cap margin (**Supplementary Figure 1**).

The liquid cultures were grown by transferring mycelia + agar slices to sterilized malt extract broth in ventilated culture flasks and incubated at 26°C (*P. cubensis*) or 21°C (*P. allenii* and *P. cyanescens*) for approximately 21 days. The bulk substrate, consisting of 600 g of coconut coir, 280 g of vermiculite, and 124 g of gypsum at field capacity for moisture, once fully colonized, was subjected to fruiting conditions. The fruiting conditions consisted of a 12-hour photoperiod at a Kelvin color temperature of approximately 6,400 and a decrease in temperature to 21°C. The bag of bulk substrate, which had a 0.5-micron filter patch to allow for gas exchange, acted as a miniature greenhouse, maintaining moisture and humidity within the bag to allow for sporocarp formation. The sporocarps were harvested approximately 14 days after being subjected to fruiting conditions.

The mushrooms and grain mycelia samples were dehydrated and ground, whereas liquid mycelial cultures were collected from the cell culture flasks and freeze-dried using Toption and Harvest Right Vacuum Freeze-Dryers. For both Orbitrap Exploris and TSQ Altis, the samples were weighed out to 25 mg± 5 mg, dissolved in 5 mL of methanol, and mixed at 1,800 rpm in a Benchmark Scientific BenchMixer™ multi-tube vortexer, followed by a 5-min sonication. The samples were then centrifuged at 3,100 *x* g for 15 min at −8°C. The supernatant was collected, and the material was once again extracted following the same procedure, after which the supernatants were pooled.

### Screening of targeted compounds

For the targeted analysis, samples were analyzed using a TSQ Altis Triple Quadrupole Mass Spectrometer coupled with UHPLC equipped with heated electron spray ionization (H-ESI) for data acquisition. Data points were collected for 25 min for each sample. The scan was carried out by way of selected reaction monitoring (SRM) with positive polarity, collecting 13.3 points per peak. For each compound, the following parameters were included in the SRM: retention (RT), precursor [mass-to-charge ratio (m/z)], product(s) (m/z), collision energy (V), and RF lens (V). The resolution for both Q1 and Q3 was 0.7 FWHM (full width at half maximum). A spiked sample was prepared along with every sample run as part of the quality control process for analytical testing. Each sample run included three replicates for a sample (A, B, and C) and also a procedural blank consisting of methanol and internal standards at the same dilution, which was subjected to the same extraction procedure, and a solvent blank consisting of methanol only. The level of detection (LOD) was 0.00002 mg/g and the limit of quantification (LOQ) varied across the standardized compounds.

The fungal samples were given a unique ID (**Supplementary Table 1**) along with a certificate of analysis for standardized compounds, including: alpha-glycerylphosphorylcholine (AGPC), arginine (ARG), baeocystin (BAEO), carnitine (CARN), choline (CHOL), ergothioneine (ERGO), glutamate (GLU), glutamine (GLN), histidine (HIS), methoxy-tryptamine (MTRY), *N*-acteylglucosamine-anhydro (NAGA), nicotinamide (NICO), nicotinic acid (NICA), norbaeocystin (NORB), pantothenic acid (PANT), phenylethylamine (PEA), psilocin (PSC), psilocybin (PSB), serotonin (SERO), trimethylglycine (TMG), trimethyllycine (TML), tryptophan (TRP), aeruginascin (AERU), norpsilocin (NORP), and 4-hydroxy-*N,N,N*-trimethyltryptamine (4HTT). The samples were run as three technical replicates (*n* = 3) and averaged. All the targeted data are presented in mg/g.

### Screening of untargeted compounds

For the untargeted analysis, the samples were analyzed using an Orbitrap Exploris 120 equipped with H-ESI with a full-scan method. The data points were collected for 22 min for each sample. The resolution of the mass spectrometer was set to 15,000, with a scan range of 80 m/z–700 m/z. The instrument was set to positive polarity. Each sample run included three replicates for a sample (A, B, and C), in addition to a procedural blank and a solvent blank.

The raw data files were returned from Xcalibur™ (Thermo Fisher Scientific), which were then converted to an mzML file format using a ProteoWizard MS converter and loaded into MZmine2.53. The mass detection of raw data was done through a local minima search with the noise level set to 4.0E + 06, and peaks with retention times (RTs) between 0 min and 12 min were included. Throughout data preprocessing, an m/z tolerance of 0.005 was set. An automated data analysis pipeline (ADAP) chromatogram builder was used to generate the chromatograms. The minimum threshold was set to 3.0E + 06, and the minimum peak intensity was set to 4.0E + 06. The chromatogram deconvolution consisted of visualizing the individual spectral scans to capture unique peaks, changing the chromatographic threshold to 20%, the minimum RT range to 0.05 min, the minimum relative height to 15%, the minimum absolute height to 5.0E + 06, and the minimum ratio of the peak top edge to 1. An isotopic peak grouper was used with a maximum charge of 1 and a monotonic shape was applied to the peak. A join aligner allowed buckets to be made to create a two-dimensional aligned chromatogram, and gap-filling was conducted using the peak finder function with a 5.0% intensity tolerance and 0.05 RT tolerance. The joined and gap-filled chromatogram was then exported to CSV with the peak ID, RT, and m/z value. Data pruning consisted of removing the features that did not reach the minimum threshold of 4.0E + 06 in samples or that were abundantly present in the procedural blanks. Peak annotation was performed in Xcalibur using the compound discovery application, where percentage identity scores were created based on MS2 spectra when compared with internal and external compound databases.

### Statistical analysis

Principal component analysis (PCA) is an unsupervised clustering method that reduces the dimensionality of multivariate data while preserving most of their variance. All samples were collected from a unique fruiting bag, grain spawn bag, or mycelial culture, and were assigned a unique internal lot number and barcode. Studying the extracted fungal material using the H-ESI full scan revealed a large cloud of mass features, the data structure of which was parsed from the nose using PCA. On generating the PCA plots, all scree plots (data not shown) were observed to confirm that inflection occurs after PC1 and PC2. The analysis of the mycelia, grain spawn mycelia, and fruiting bodies, and also the comparisons between strains of *P. cubensis* fruiting bodies, used an unsupervised clustering method along with ellipses, and all data matrices were Pareto scaled before analysis.

A univariate analysis of indole alkaloid and minor compound content was used to determine the relative difference in mycochemicals among the groups and further evaluate any emerging correlations in the dataset. ANOVA and Tukey’s honestly significant difference (HSD) were used to generate *F*-statistics, fold differences, and to identify significant differences among the groups based on targeted or untargeted compounds. For all data groups, a Shapiro–Wilk test was used to ensure normality assumptions were met, and all data groups contained ≥ five replicates.

## Results

### Targeted analysis of *Psilocybe* mycelium, grain mycelium, and fruiting bodies

**Figure 2:**
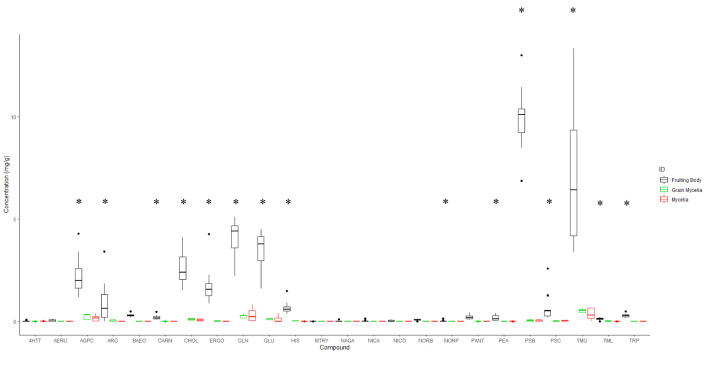
Box plot representing the accumulation of targeted compounds analyzed using TSQ Altis as determined by a standard curve. The data were collected from 14 fruiting bodies, seven mycelia and six grain mycelia samples. * (*p* < 0.05) in ANOVA and Tukey’s HSD. 4HTT, 4-hydroxytryptamine; AERU, aeruginascin; AGPC, α-glycerylphosphorylcholine; ARG, arginine; BAEO, baeocystin; CARN, carnosine; CHOL, choline; ERGO, ergotheioneine; GLN, glutamine; GLU, glutamic acid; HIS, histidine; MTRY, methoxytryptamine; NAGA, N-acetylglucosamine-anhydro; NICA, nicotinamide; NICO, nicotinic acid; NORB, norbaeocystin; NORP, norpsilocin; PANT, panthotenic acid; PEA, phenylethylamine; PSB, psilocybin; PSC, psilocin; TMG, trimethylglycine; TML, trimethyllysine; TRP, tryptophan.

The univariate analysis of indole alkaloids and other fungal metabolites revealed that fruiting bodies preferentially accumulate aeruginascin, alpha-glycerophosphocholine, carnitine, choline, ergothioneine, glutamate, glutamine, histidine, norbaeocystin, pantothenic acid, phenylethylamine, psilocin, psilocybin, trimethylglycine, trimethyllysine, and tryptophan (**Figure 2**) when compared with mycelium and grain mycelium. The average psilocybin (± SE) content in fruiting bodies was 9.913 mg/g ± 0.389 mg/g, 0.041 mg/g ± 0.014 mg/g for mycelia, and 0.047 mg/g ± 0.023 mg/g for grain mycelium samples. In contrast, no significant differences were found in the concentrations of 4-hyrdoxy-*N,N,N-*trimethyltryptamine, arginine, methoxy-tryptamine, *N-*acetyl glucosamine anhydro, nicotinamide, nicotinic acid, and norpsilocin between fruiting bodies and mycelia (**Supplementary Table 2**).

**Figure 3:**
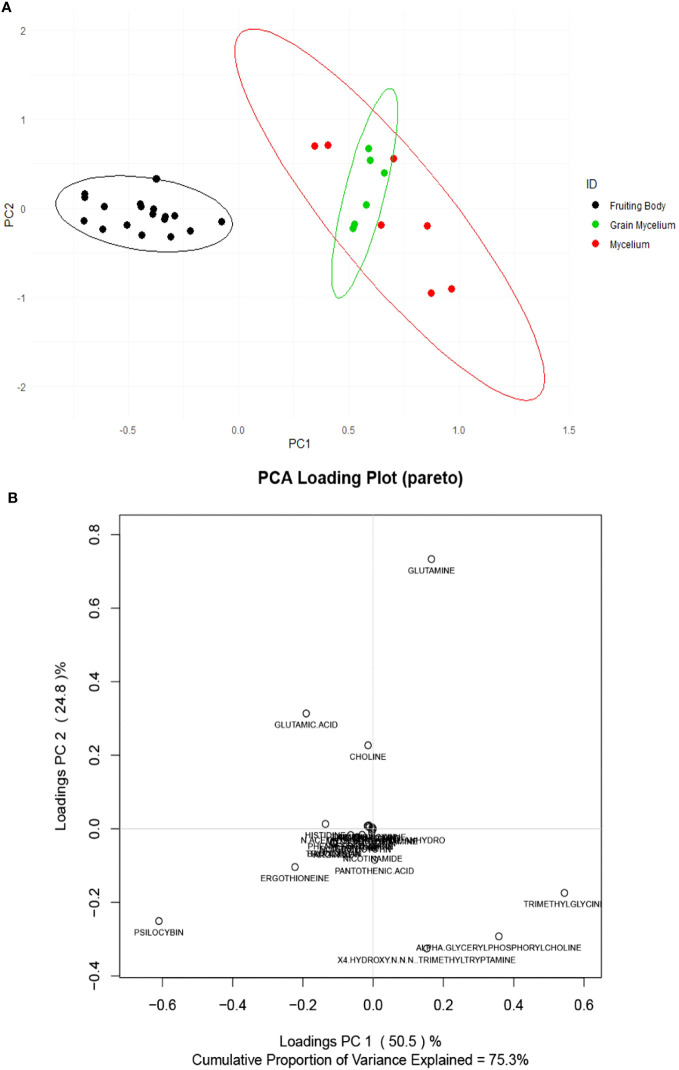
Score **(A)** and loading **(B)** plots of *Psilocybe cubensis* fruiting bodies (scaled and unsupervised). The mycelium (red) consisted of seven *Psilocybe* mycelium samples of *Psilocybe cyanescens, Psilocybe allenii*, and *P. cubensis*. The grain mycelium (green) consisted of six samples of *P. cubensis* and *P. cyanescens.* The fruiting bodies (black) consisted of 18 samples from *P. cubensis*. The samples were extracted and quantified using TSQ Altis, and the data were averaged over three technical replicates (*n* = 3) for each data point.

The preparations of *P. cubensis* mycelium and grain mycelium were compared for their targeted compound content. Targeted analysis revealed the fruiting body chemistry to be chemically distinct from that of mycelium and grain mycelium, as indicated in a PCA score plot (**Figure 3A**). The loading plot (**Figure 3B**) reveled psilocybin influencing the clustering of fruiting bodies while α-GPC and trimethylglicine the mycelia. However, based on targeted data, no significant difference was found between grain mycelia and mycelia. Principal components 1 and 2 captured 75.3% of the total variation in the dataset. Hierarchical cluster analysis of the samples, using an unweighted pair group method with arithmetic mean (UPGMA) and a Euclidean distance function, revealed similar patterns in terms of the relatedness of samples in targeted compounds data and also of the relatedness of the compounds (**Supplementary Figure 2**).

Psilocybin is present in mycelia and grain mycelia, but the isolation of indole alkaloids from fungal materials will be most effective by the extraction of fruiting bodies. There was no significant difference in psilocin, norpsilocin, and aeruginascin content among the life stages. An ANOVA of the individual compounds revealed that levels of alpha-glycerylphosphorylcholine (α-GPC), *N-*acetylglucosamine, and trimethylglycine accumulation were found to be higher in mycelia and grain mycelia than in fruiting bodies, according to Pareto-scaled data (*p* < 0.05). It is known that α-GPC is a class of phosphocholines that delivers choline to the brain and is a precursor to acetylcholine, and it is also a neurotransmitter and modulator involved in arousal, attention, memory, and motivation (Sigala et al., 1992). In addition, α-GPC has been implicated in enhanced physical motor performance (Bellar et al., 2015; Marcus et al., 2017) and enhanced cognition (Lee et al., 2017), while *N-*acetylglucosamine is the monomeric unit of the polymer chitin which is abundant in medicinal mushroom preparations (Chen et al., 2010). Finally, trimethylglycine (betaine) is essential to the methylation of methionine in the homocysteine methyltransferase cycle (Brütting et al., 2021) and also to cellular osmoregulation (Lever and Slow, 2010). Considering the low psilocybin content of mycelia and the accumulation of beneficial fungal metabolites in them, *Psilocybe* mycelia may be good candidates for mycotherapeutic development.

### Untargeted analysis of *Psilocybe* mycelium, grain mycelium, and fruiting bodies

**Figure 4:**
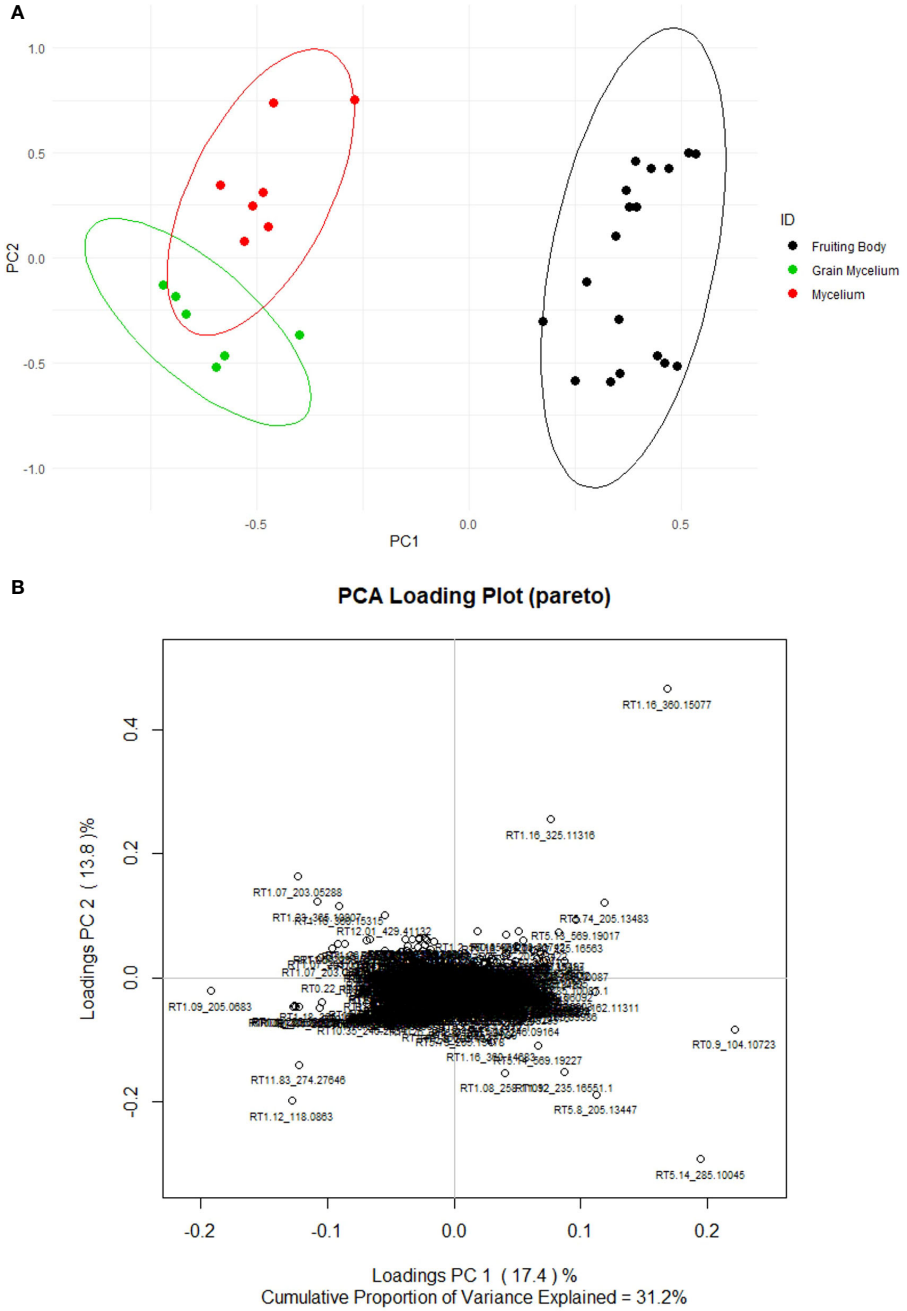
Score **(A)** and loading **(B)** plots of *Psilocybe* fruiting bodies (non-scaled and unsupervised). The mycelium (red) consisted of seven *Psilocybe* mycelium samples of *Psilocybe cyanescens, Psilocybe allenii*, and *Psilocybe cubensis*. The grain mycelium (green) consisted of six samples of *P. cubensis* and *P. cyanescens.* The fruiting bodies (black) consisted of 18 samples from *P. cubensis*. The principal components were determined based on the loaded 978 mass features returned by Orbitrap Exploris H+ESI. The mass features were pruned with method and procedural blanks and then they were Pareto scaled.

The preprocessing of Orbitrap Exploris 120 full-scan data yielded an aligned data matrix consisting of 978 mass features across samples of all types. Each feature is denoted by an RT and m/z (**Figure 4**). Only 31.2% of variability in the dataset was captured by principal components 1 and 2. However, the enhanced separation of individual samples was observed within and among the groups. The fruiting body chemistry is different from that of mycelium and grain mycelium (**Figure 4A**). A greater separation of mycelium and grain mycelium confidence was observed; however, these differences were insubstantial. Hierarchical cluster analysis using an unweighted pair group with arithmetic mean (UPGMA) of samples and a Euclidean distance function revealed similar patterns in terms of the relatedness of the samples in targeted compounds data and also of the relatedness of metabolites (**Supplementary Figure 3**). The clustering revealed that fruiting bodies, mycelia, and grain mycelia exhibit a unique suite of fungal metabolites.

The separation within fruiting bodies appeared to be influenced by the mass features RT5.14_285.10045 and RT1.16_360.15077, which are precursor peaks for psilocybin and trehalose, respectively, as determined by MS2 spectra. These mass features appeared to polarize the data in the PC1 loadings plot when the mycelia and fruiting bodies were compared. The further isolation and quantification of fungal sugars such as α-glucans and β-glucans (Pérez-Vendrell et al., 1995), and of monosaccharides and disaccharides may be beneficial in future studies. These results may further expand on the potential inverse relationship between *Psilocybe* psilocybin and carbohydrate content.

An analysis of *Morchella* (morel) based on UPLC-ESI-MS/MS data revealed that mycelium and fruiting bodies also displayed different metabolomes with upregulated and downregulated metabolic pathways (Yang et al., 2021). A comprehensive study of *Isaria cicadae* (cordyceps) revealed that growth media and environmental conditions influence mycelial and fruiting body metabolomes, such that fruiting bodies may accumulate higher antioxidant compounds due to their exposure to oxygen (He et al., 2019). This pattern may also hold true for *Psilocybe* species and should be considered in the further development of research on psychedelic mushrooms.

## Discussion

Although the metabolites from the indole alkaloid synthesis pathway have an established pharmacology, their bioactivity is not limited to this class of compounds, as other selected fungal metabolites, amino acids, lipids, and sugars may positively or negatively modulate indole alkaloid involvement in various pathologies (Brown and Gordon, 2003; Evidente et al., 2014). The crude extract preparations of *Psilocybe* mushrooms may exhibit synergistic effects when compared with pure psilocybin due to the binding of the orthosteric site on 5-HT_2A_ receptors (Glatfelter et al., 2022), or the potential affinity for off-target receptors, which may be additive, such as inhibiting the degradation or reuptake of endogenous serotonin. Allosterism has been described for serotonin receptors (Fasciani et al., 2020), and this site may be targeted by metabolites that exist in fruiting body or mycelial extracts. This may be similar to the relationship between Δ^9^-tetrahydrocannabinol (THC) and cannabidiol (CBD) from *Cannabis*, where CBD is a negative allosteric modulator of THC at the cannabinoid receptor 1 (Laprairie et al., 2015). The synergism between the mushroom's active compound and other fungal metabolites may enhance the therapeutical effect described with pure psilocybin. Another example is the anti-depressant synergistic effects of active compounds from *Hypericum perforatum* (St. John’s Wort) (Simmen et al., 2001). Hypericin and other active compounds showed inhibitory activity at corticotrophin-releasing factor receptors as well as gamma-aminobutyric acid type A GABA_A_, serotonin, and opioid receptors.

Active sites of tryptamine derivatives in *Psilocybe* spp. match those of serotonin receptors (5-HT_2A_) (Almaula et al., 1996). The binding of 5-HT_2A_ G-protein-coupled receptors (GPCRs) and the resulting hallucinogenic effects elicited by psilocybin/psilocin are determined by distinct downstream signaling events, biased agonism (Karaki et al., 2014; Pottie et al., 2020), the binding of off-target receptors, and the formation of heterocomplexes between the 5-HT_2A_ and D_2L_ and mGlu2/3 receptors (Albizu et al., 2011; Inserra et al., 2021). The benefits of psilocybin are dependent on a delicate cascade of signaling spurred by the activation of GPCRs and further recruitment of G subunits and β-arrestins (López-Giménez and González-Maeso, 2018). The effects of *Psilocybe* metabolite allosteric interactions at the level of the receptor and potential synergism by binding of dopamine and glutamate receptors have yet to be investigated. The cumulative effects of crude extracts substantiate why traditional medicine practitioners believe these preparations have better therapeutic activity than the compounds isolated from them (Koltai and Namdar, 2020).

The levels of indole alkaloids psilocybin, baeocystin, norbaeocystin, and tryptophan were significantly higher in fruiting bodies than in the mycelium. Overall, mycelium and grain mycelium could not be distinguished from each other in the targeted and untargeted analysis; however, fruiting body chemistry was found to be significantly different in the univariate analysis. The retention times and mass-to-charge ratios were used to identify dominant peaks in MS2 spectra; however, many mass features remain unknown in this analysis. As *Psilocybe* preparations become more prevalent in the recreational and psychedelic-assisted therapy space, a well-assembled fungal mass spectra library to identify potential bioactive or harmful compounds will be a useful tool for researchers and regulators. Mycelia and grain mycelia cultures are easy to expand, and fungal metabolites in them remain in high-enough proportions, with the level of psilocybin accumulation remaining low. In this study we provide the analysis necessary to propose *Psilocybe* mycelia as a non-intoxicating mycotherapeutic with chemistries significantly different than those of fruiting bodies.

## Data availability statement

The data presented in the study are deposited in the Center for Open Science repository and can be accessed at https://osf.io/jctyk.

## Author contributions

AW: Conceptualization, Data curation, Formal Analysis, Investigation, Writing – original draft. MB: Data curation, Investigation, Methodology, Writing – original draft. SN: Data curation, Investigation, Methodology, Writing – original draft. JA: Conceptualization, Project administration, Supervision, Writing - review & editing. SS: Conceptualization, Funding acquisition, Project administration, Resources, Supervision, Writing – review & editing.

## Supplementary material

Supplementary Figure 1 Fully colonized fruiting block of *P. cubensis* pre-harvest.

Supplementary Figure 2 Heat map of fruiting body (*n* = 18), mycelial (*n* = 7), and grain mycelial samples (*n* = 6) and known targeted compounds. The Euclidean distance function was used to generate dendrograms, and columns and rows were clustered using an unweighted pair group with arithmetic mean (UPGMA) in R.

Supplementary Figure 3 Heat Map of Fruiting Body (n=18), Mycelial (n=7) and Grain Mycelial samples (n=6) and untargeted compounds. The Euclidean distance function was used to generate dendrograms, and columns and rows were clustered using an unweighted pair group with arithmetic mean (UPGMA) in R.
