# Supplementary figures and images for: Exploring Psilocybe spp. mycelium and fruiting body chemistry for potential therapeutic compounds
Source: Front Fungal Biol. 2023 Nov 29;4:1295223. doi: 10.3389/ffunb.2023.1295223 (PMC10716206; doi:10.3389/ffunb.2023.1295223)

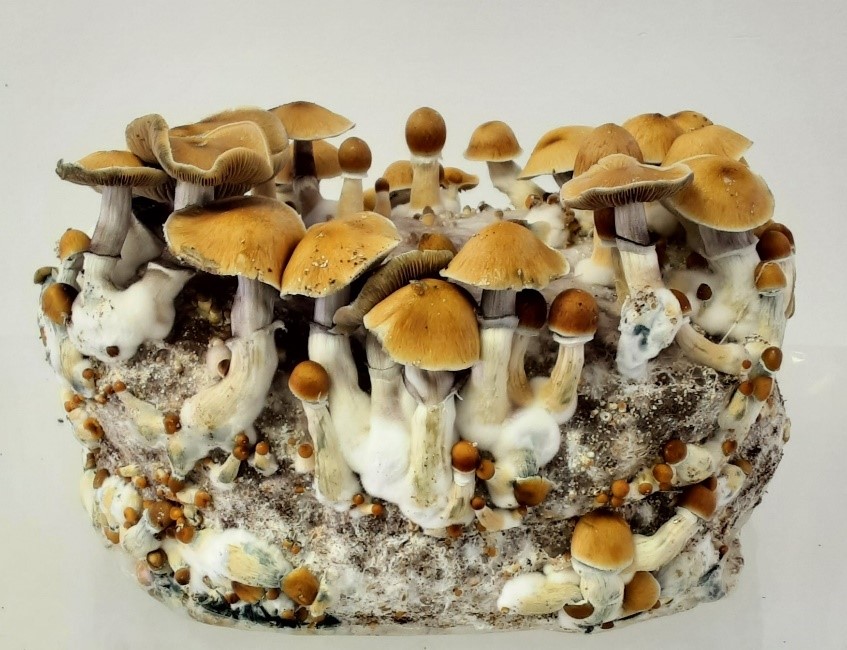

Supplement: Supplementary Figure 1 — Fully colonized fruiting block of P. cubensis pre-harvest. [file Image_1.jpeg]

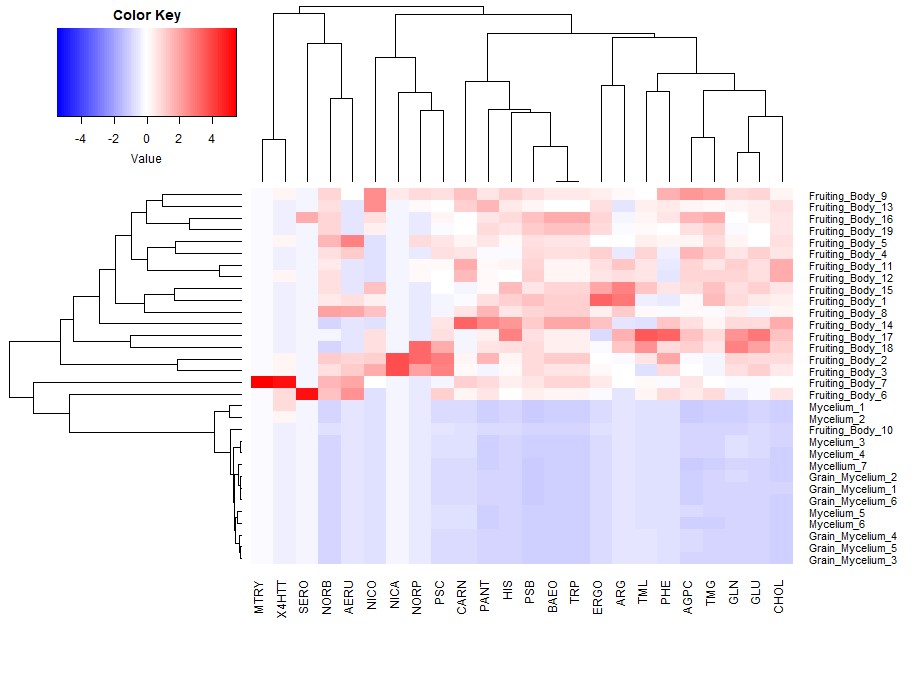

Supplement: Supplementary Figure 2 — Heat map of fruiting body (n = 18), mycelial (n = 7), and grain mycelial samples (n = 6) and known targeted compounds. The Euclidean distance function was used to generate dendrograms, and columns and rows were clustered using an unweighted pair group with arithmetic mean (UPGMA) in R. [file Image_2.jpeg]

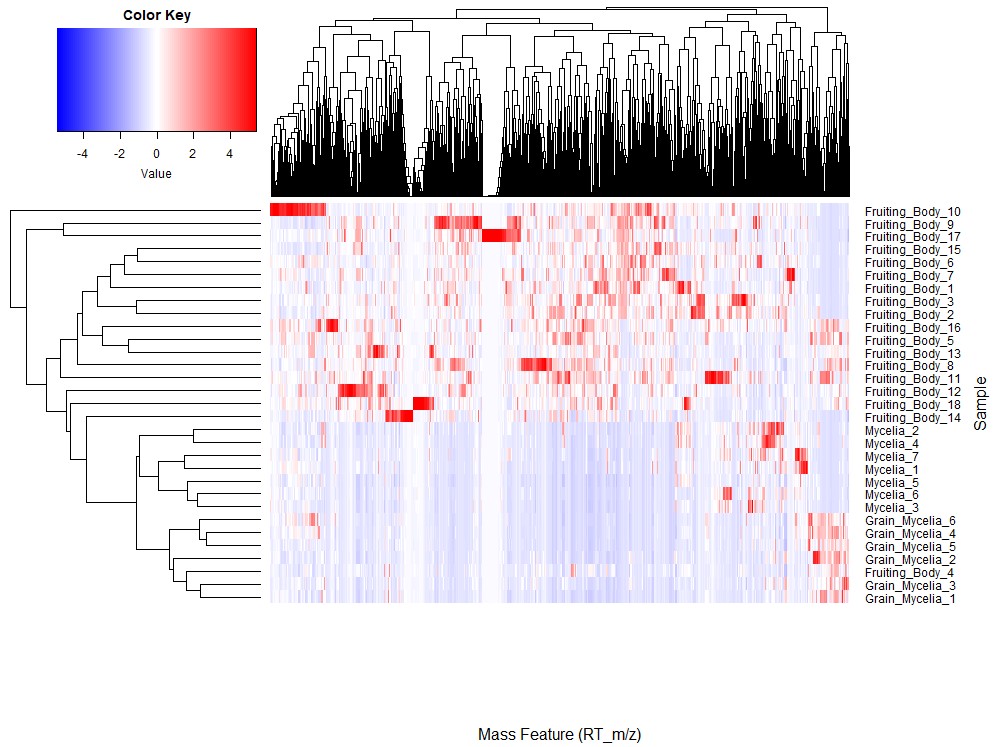

Supplement: Supplementary Figure 3 — Heat Map of Fruiting Body (n=18), Mycelial (n=7) and Grain Mycelial samples (n=6) and untargeted compounds. The Euclidean distance function was used to generate dendrograms, and columns and rows were clustered using an unweighted pair group with arithmetic mean (UPGMA) in R. [file Image_3.jpeg]
