# Supplementary material for: Exploring Psilocybe spp. mycelium and fruiting body chemistry for potential therapeutic compounds
Source: Front Fungal Biol. 2023 Nov 29;4:1295223. doi: 10.3389/ffunb.2023.1295223 (PMC10716206; doi:10.3389/ffunb.2023.1295223)
Supplement: Supplementary file 4 [file Table_1.docx]

| **ID** | **Species** | **Strain ID** | **Culture type** |
| --- | --- | --- | --- |
| 2431_BPAMUL_FB | *Psilocybe cubensis* | BP-A-MUL | Fruiting Body |
| 2603_BPSTAR_FB | *Psilocybe cubensis* | BP-STAR | Fruiting Body |
| 2453_PAZ1_MP | *Psilocybe allenii* | P-AZ-1 | Liquid culture mycelium |
| 2441_BPAMUL_MP | *Psilocybe cubensis* | BP-A-MUL | Liquid culture mycelium |
| 2459_PCY1_MP | *Psilocybe cyanescens* | P-CY-1 | Liquid culture mycelium |
| 2443_BPAMUL_MP | *Psilocybe cubensis* | BP-A-MUL | Liquid culture mycelium |
| 2446_5015B1_FB | *Psilocybe cubensis* | 5015-B.1 | Fruiting Body |
| 2432_BPAMUL_FB | *Psilocybe cubensis* | BP-A-MUL | Fruiting Body |
| 2547_BPSTAR_GM | *Psilocybe cubensis* | BP-STAR | Grain spawn mycelium |
| 2481_PAZ1_MP | *Psilocybe allenii* | P-AZ-1 | Liquid culture mycelium |
| 2552_BPSTAR_GM | *Psilocybe cubensis* | BP-STAR | Grain spawn mycelium |
| 2458_PCY1_MP | *Psilocybe cyanescens* | P-CY-1 | Liquid culture mycelium |
| 2558_BPSTAR_GM | *Psilocybe cubensis* | BP-STAR | Grain spawn mycelium |
| 2534_PAZ1_GM | *Psilocybe allenii* | P-AZ-1 | Grain spawn mycelium |
| 2455_PAZ1_MP | *Psilocybe allenii* | P-AZ-1 | Liquid culture mycelium |
| 2574_LKMUL_FB | *Psilocybe cubensis* | LK-MUL | Fruiting Body |
| 2540_PAZ1_GM | *Psilocybe allenii* | P-AZ-1 | Grain spawn mycelium |
| 2528_5015B1_FB | *Psilocybe cubensis* | 5015-B.1 | Fruiting Body |
| 2473_BPASTAR_FB | *Psilocybe cubensis* | BP-A-STAR | Fruiting Body |
| 2483_LKMUL_FB | *Psilocybe cubensis* | LK-MUL | Fruiting Body |
| 2571_LKMUL_FB | *Psilocybe cubensis* | LK-MUL | Fruiting Body |
| 2477_AVMUL_FB | *Psilocybe cubensis* | AV-MUL | Fruiting Body |
| 2471_BPASTAR_FB | *Psilocybe cubensis* | BP-A-STAR | Fruiting Body |
| 2531_PAZ1_GM | *Psilocybe allenii* | P-AZ-1 | Grain spawn mycelium |
| 2522_5015B1_FB | *Psilocybe cubensis* | 5015-B.1 | Fruiting Body |
| 2579_BPSTAR_FB | *Psilocybe cubensis* | BP-STAR | Fruiting Body |
| 2600_BPSTAR_FB | *Psilocybe cubensis* | BP-STAR | Fruiting Body |
| 2621_BPAMUL_FB | *Psilocybe cubensis* | BP-A-MUL | Fruiting Body |
| 2475_AVMUL_FB | *Psilocybe cubensis* | AV-MUL | Fruiting Body |
| 2516_BPASTAR_FB | *Psilocybe cubensis* | BP-A-STAR | Fruiting Body |
| 2414_AVMUL_FB | *Psilocybe cubensis* | AV-MUL | Fruiting Body |
| 2591_BPSTAR_FB | *Psilocybe cubensis* | BP-STAR | Fruiting Body |

**Supplementary Table 1**: Sample Data with unique ID, Species name, Strain ID and Culture Type used in targeted and untargeted analysis.
