# Supplementary material for: Exploring Psilocybe spp. mycelium and fruiting body chemistry for potential therapeutic compounds
Source: Front Fungal Biol. 2023 Nov 29;4:1295223. doi: 10.3389/ffunb.2023.1295223 (PMC10716206; doi:10.3389/ffunb.2023.1295223)
Supplement: Supplementary file 5 [file Table_2.docx]

| **Compound** | **Fold Change Mycelia vs Fruiting body** | **Fold Change Grain Mycelia vs Fruiting Body** |
| --- | --- | --- |
| 4HTT | -0.0089 (n.s) | -0.0122 (n.s) |
| AERU | -0.05362** | -0.05362* |
| AGPC | -2.121*** | -2.035*** |
| ARG | -0.916* | -0.877 (n.s) |
| BAEO | -0.2897*** | -0.2897*** |
| CARN | -0.194*** | -0.199*** |
| CHOL | -2.566*** | -2.593*** |
| ERGO | -1.661*** | -1.670*** |
| GLU | -3.356*** | -3.344*** |
| GLN | -3.816*** | -3.866*** |
| HIS | -0.659*** | -0.646*** |
| MTRY | -0.00085 (n.s) | -0.00085 (n.s) |
| NAGA | -0.00753 (n.s) | -0.00753 (n.s) |
| NICO | -0.0284 (n.s) | -0.0257 (n.s) |
| NICA | -0.0179 (n.s) | -0.0179 (n.s) |
| NORB | -0.07*** | -0.07*** |
| NORP | -0.0231 (n.s) | -0.0231 (n.s) |
| PANT | -0.189*** | -0.196*** |
| PEA | -0.178** | -0.175* |
| PSC | -0.634* | -0.645* |
| PSB | -9.867*** | -9.873*** |
| TMG | -6.599*** | -6.455*** |
| TML | -0.0115*** | -0.106*** |
| TRP | -0.282*** | -0.282*** |

**Supplementary Table 2**: Comparison of fold changes in targeted compounds between *Psilocybe* Mycelia and Grain Mycelia vs Fruiting bodies. A linear ANOVA was carried out for each compound and Tukey HSD performed for fold changes and significance values. (*) p < 0.05, (**) p < 0.01, (**) p , 0.001
